# GABA_A_-Mediated Inhibition Modulates Stimulus-Specific Adaptation in the Inferior Colliculus

**DOI:** 10.1371/journal.pone.0034297

**Published:** 2012-03-29

**Authors:** David Pérez-González, Olga Hernández, Ellen Covey, Manuel S. Malmierca

**Affiliations:** 1 Auditory Neurophysiology Unit, Institute of Neuroscience of Castilla y León, University of Salamanca, Salamanca, Spain; 2 Faculty of Medicine, Department of Cell Biology and Pathology, University of Salamanca, Salamanca, Spain; 3 Department of Psychology, University of Washington, Seattle, Washington United States of America; Claremont Colleges, United States of America

## Abstract

The ability to detect novel sounds in a complex acoustic context is crucial for survival. Neurons from midbrain through cortical levels adapt to repetitive stimuli, while maintaining responsiveness to rare stimuli, a phenomenon called stimulus-specific adaptation (SSA). The site of origin and mechanism of SSA are currently unknown. We used microiontophoretic application of gabazine to examine the role of GABA_A_-mediated inhibition in SSA in the inferior colliculus, the midbrain center for auditory processing. We found that gabazine slowed down the process of adaptation to high probability stimuli but did not abolish it, with response magnitude and latency still depending on the probability of the stimulus. Blocking GABA_A_ receptors increased the firing rate to high and low probability stimuli, but did not completely equalize the responses. Together, these findings suggest that GABA_A_-mediated inhibition acts as a gain control mechanism that enhances SSA by modifying the responsiveness of the neuron.

## Introduction

Neuronal adaptation is ubiquitous in the brain. However, most forms of adaptation described in previous studies depend only on the activation history of the neuron [1]–[4]. Stimulus-specific adaptation (SSA) is a phenomenon observed in single neurons that consists of a progressive decline of the response to an often-repeated stimulus (called the standard), while maintaining full responsiveness to those stimuli with a low probability of occurrence (called the deviants). SSA has been found at different levels of the auditory system, from the midbrain to the cortex [5]–[10]. SSA can be thought of as a filtering mechanism at the cellular level that depends on the history of stimulation and prevents neurons from responding to continuously repeated sounds [8], [11], [12]. Therefore, SSA may contribute to auditory scene analysis [13], attention [14], [15] and novelty or change detection [16]–[18].

The basic properties of SSA have been studied in detail [5], [6], [8], [19], [20], but the neuronal mechanisms that generate SSA are currently unknown. SSA depends on the physical contrast between standard and deviant stimuli, the probability of occurrence of the deviant stimulus, and the time intervals between stimuli. Previous studies have suggested that SSA first appears in cortical circuitry, and linked it with auditory memory and recognition of acoustic objects [21], [22]. Despite the increasing number of studies at multiple levels in recent years, it is not clear whether SSA is generated in one structure and then propagated to the others or whether it is a general mechanism that operates independently in each structure.

The inferior colliculus (IC) is the midbrain auditory center where nearly all ascending pathways converge [23] before sending information on to the auditory cortex via the thalamus. Thus, the IC is a key processing centre in the auditory pathway. Much of this processing is mediated by inhibition and the interplay between excitation and inhibition that takes place on its neurons, e.g. [24]. For example, GABAergic inhibition has been found to shape temporal, spectral and binaural properties in IC neurons [24]–[30] and even generate new forms of selectivity [31], [32]. Furthermore, intracellular recordings show that excitation in IC neurons is often followed by long-lasting hyperpolarization, probably due to synaptic inhibition [33], [34].

Here we test the hypothesis that synaptic inhibition, in particular the inhibition mediated by GABA_A_ receptors, plays a role in the generation of SSA in the IC of the rat. For this purpose we recorded from well-isolated single units in the IC using tungsten electrodes attached to multibarrel glass pipettes. Recordings were obtained before, during and after microiontophoretic application of the GABA_A_ receptor antagonist gabazine, while presenting sounds in an oddball paradigm [8]. In this paradigm a sequence is constructed so that in each trial one of two stimuli (in our case, pure tones of different frequencies, *f_1_* and *f_2_*) is presented, but each stimulus has a different probability of occurrence. The stimulus presented with the higher probability is called the standard, while the low probability stimulus is called the deviant. A second sequence containing the same stimuli is presented, but their probabilities are switched so that the deviant frequency becomes the standard and vice versa. We found that synaptic inhibition via GABA_A_ receptors does, in fact, modulate SSA, but that the firing rate, latency and discharge pattern of IC neurons to high and low probability stimuli remain distinct even when GABA_A_ receptors are blocked.

## Results

In this study we recorded from 46 highly adapting single units in the cortical subdivisions of the IC in rats. When a neuron was isolated, we recorded spike times in response to stimuli presented using the oddball protocol, obtaining a set of baseline data. We then applied gabazine and continued to present the sequences. Data were collected at regular time intervals during gabazine application and again following gabazine until the responses recovered to baseline levels. Our main results show that the application of gabazine has a profound effect on the magnitude of SSA and the time course of adaptation, partially restoring responses to high-probability stimuli and delaying adaptation. However, even when responses to the standard stimuli are restored, latency and discharge pattern depend on whether a given stimulus is presented as the standard or the deviant regardless of frequency.

### Effect of gabazine on response magnitude and SSA indices

**Figure 1:**
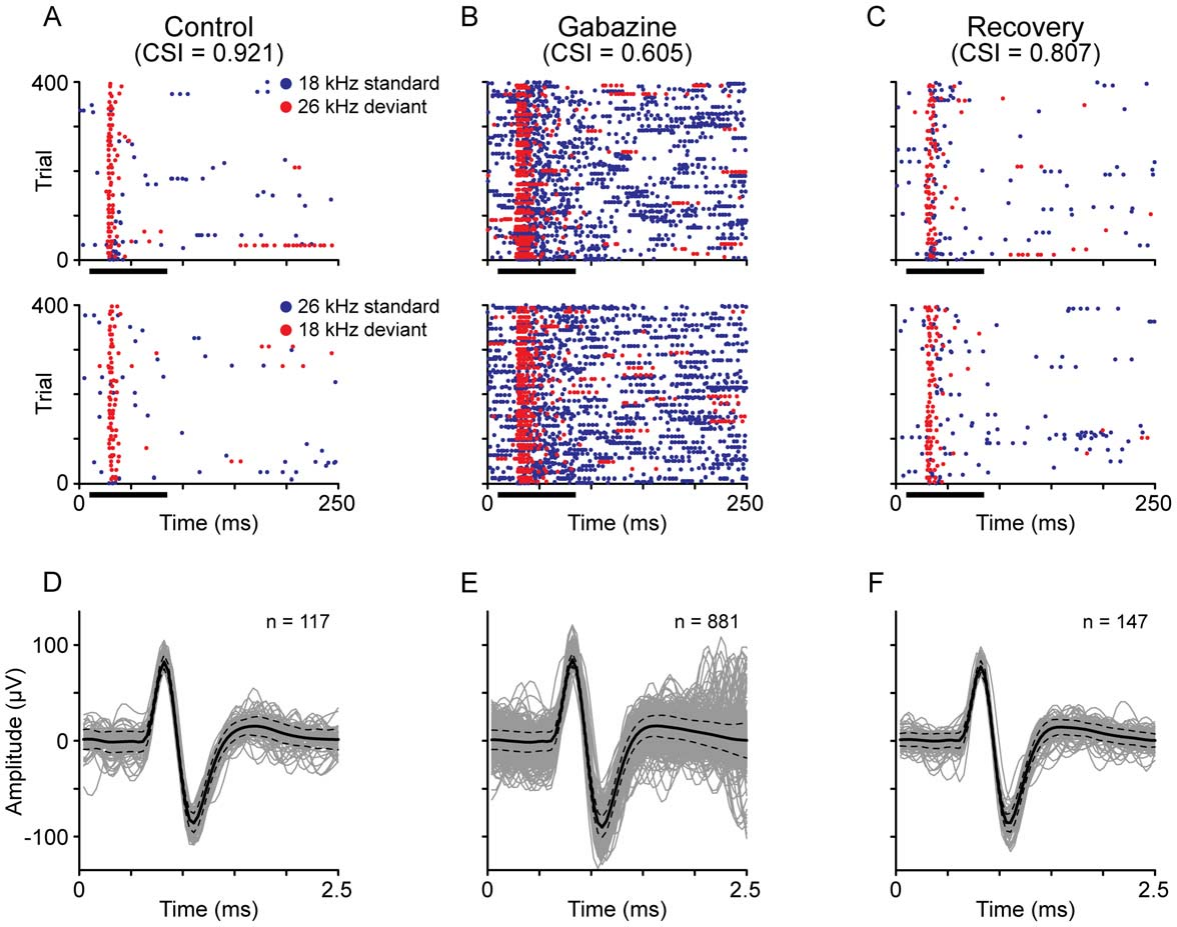
Responses of a neuron during the application of gabazine. (A–C) Dot rasters showing the typical effect of gabazine on the responses of a neuron. A sequence of 400 stimuli (trials) was played in an oddball paradigm. Each stimulus was a pure tone, at a frequency of either 18 kHz or 26 kHz (top row). 90% of the trials in the sequence consisted of an 18 kHz tone (standard, *std*) and 10% a 26 kHz tone (deviant, *dev*). To account for differences in the response due to physical characteristics of the stimuli, the probabilities were reversed (i.e. 26 kHz was the standard and 18 kHz the deviant) and the sequence played again (second row). The blue dots represent spikes evoked by the standard stimulus, while the red ones represent those evoked by the deviant stimulus. The horizontal black bar indicates the duration of the stimulus. (D–F) Waveforms of the spikes recorded from this neuron during the control (D), gabazine (E) and recovery condition (F). Despite of the increment of spikes during the application of gabazine, the shape and amplitude of the spikes remained constant, which indicates that the recordings were performed on single units. The grey traces are the individual spikes, while the black traces represent the mean (solid line) and standard deviation (dotted lines).

The application of gabazine produced a significant increase in response magnitude as seen by spikes/stimulus (Bootstrapping, 95%, c.i., confidence interval) for most neurons (41/46, for all combinations of frequency and probability). Four neurons showed a slightly decreased firing rate for some combinations, but it was not statistically different from the control rate. Only one neuron showed significantly decreased responses to all stimuli. The rate of spontaneous activity (median: 0.26 spikes/s, interquartile range IQR: 1.45; under control conditions) increased during the application of gabazine (1.72 spikes/s, IQR: 4.41), and decreased again during recovery to a rate close to that recorded under control conditions (0.41 spikes/s, IQR: 1.59). Figure 1 shows the changes in response that typically occurred during the application of gabazine.

**Figure 2:**
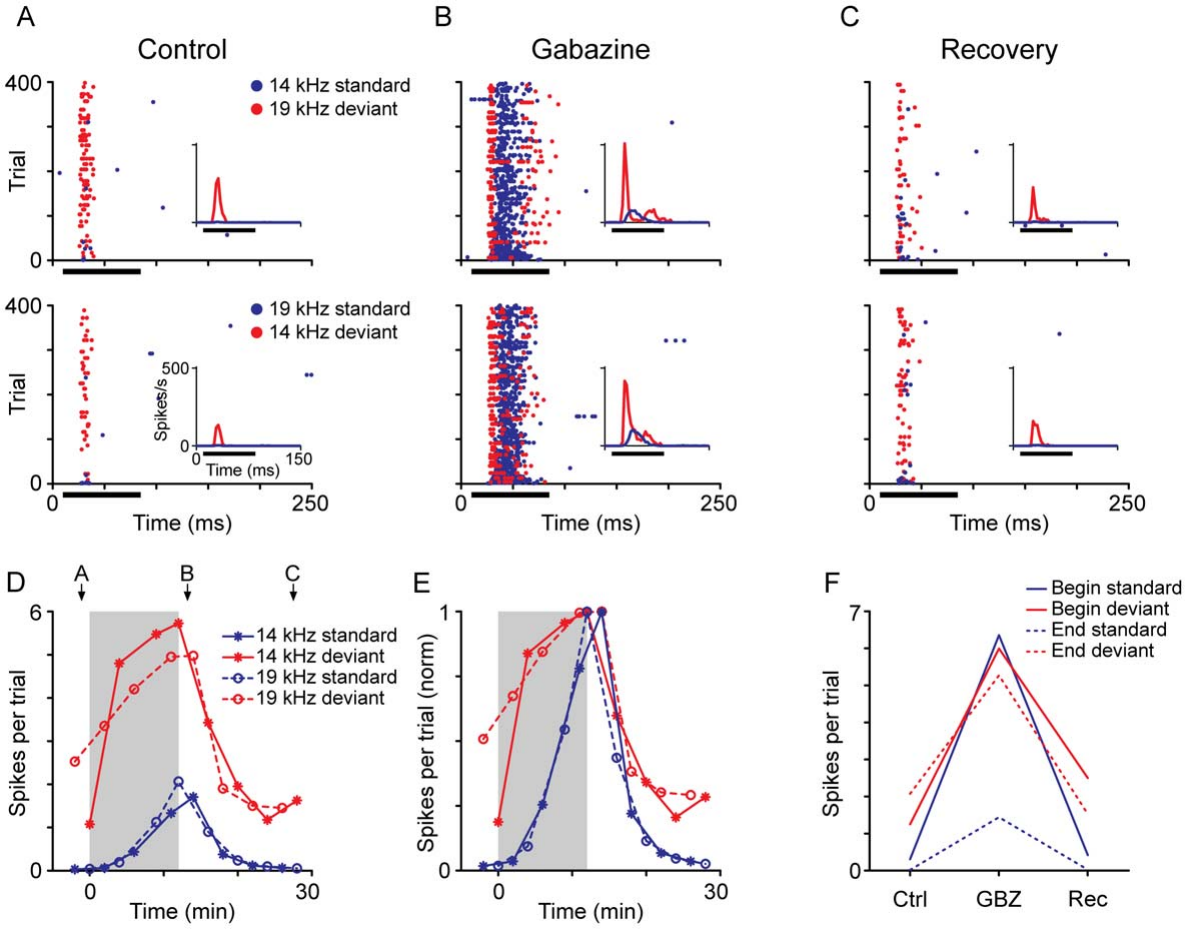
Effect of gabazine on response magnitude. (A–C) Dot rasters showing the effect of gabazine on the responses of a neuron. The protocol was the same as in Figure 1A–C, but in this case the frequencies chosen were 14 kHz and 19 kHz. The insets show a peristimulus time histogram (PSTH) of the response (3 ms bin size); the horizontal black bar indicates the duration of the stimulus. Before the application of gabazine (A), this neuron responded much more strongly to both frequencies when they were presented as the deviant than when presented as the standard, resulting in a CSI of 0.965. The application of gabazine for 12 minutes (B) increased the response to both types of stimuli, but the relative increment was larger for the standards, causing the CSI to drop to 0.480. Fifteen minutes after the end of gabazine application, the neuron's response recovered to the control level, and the CSI increased to 0.926. (D) Evolution of the response magnitude of the neuron (mean spikes per trial) shown in (A–C) in response to standards (blue lines) and deviants (red lines) during the experiment. Note that the changes are similar for both frequencies (asterisk and circles) and the main differences are due to the probability condition. The shaded background represents the application of gabazine, which starts at T = 0. The arrows indicate the times corresponding to the dot rasters in (A–C). (E) Evolution of the response magnitude of the neuron, normalized to the peak of the functions in (D). (F) Effect of gabazine on the response magnitude (mean spikes per trial) of the same neuron at the beginning of the sequence (solid lines, first 20 trials) compared with the late part of the sequence (dotted lines, last 200 trials), averaging both frequencies. Note how the effect of gabazine is smaller for the last part of the sequence for standard stimuli.

The effect of gabazine was different for standard and deviant tones. Figure 2A–C shows an example of the effect of gabazine on response magnitude and discharge pattern. Prior to gabazine application, this neuron had a larger response to deviant tones (red dots) than to standard ones (blue dots, Fig. 2A). We used the common SSA index (CSI, see *Methods*) to quantify the response to deviants and standards. This index ranges from −1 to +1, and the positive values indicate a larger response to the deviant. The response of this neuron resulted in a CSI of 0.965. Gabazine caused a significant increase in the spike counts evoked by all test stimuli regardless of frequency or probability (Fig. 2B) but spike counts remained larger for the deviant stimuli throughout the course of the experiment (Fig. 2D). However, the CSI dropped to 0.480, indicating that the deviant to standard ratio was smaller. In this and other neurons (18/46, 39%), the effect of gabazine on spike counts occurred sooner for deviant stimuli than for standard stimuli, as observed from the normalized response functions (Fig. 2E). In other neurons the effect occurred at similar times for both types of stimuli, but was almost never faster for the standards (2/46, 4%). Spike counts and CSI recovered to pre-drug values after gabazine application was terminated (Fig. 2C).

The effect of gabazine changed throughout the time-course of stimuli presentation. We analyzed separately the average spikes per stimulus presentation during the first 20 trials and the final 200 trials of the sequence (Fig. 2F). Gabazine caused spike counts evoked by both standard and deviant stimuli to increase, with response to the deviant (red) approximately the same at the beginning and at the end of the sequence. However, in the case of the standards, spike counts were much higher at the beginning of the sequence than at the end, indicating that adaptation still occurred even in the absence of GABA_A_-mediated inhibition.

**Figure 3:**
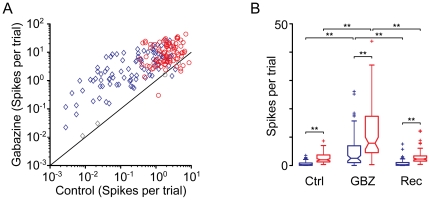
Effect of gabazine on response magnitude in the population. (A) Gabazine increased the response (spikes per trial) of almost all neurons, but the effect was different for standards (blue diamonds) than for deviants (red circles). Each symbol corresponds to one of the pair of stimuli for each neuron. Colored symbols indicate that the effect of gabazine was significant (Bootstrapping, 95% c.i.). Gray symbols represent changes that were not statistically significant. (B) Box plot showing the distribution of the mean response magnitude across the population of neurons in the control (Ctrl), gabazine (GBZ) and recovery (Rec) conditions, for deviant (red) and standard (blue) stimuli. The asterisks indicate significant differences (Friedman test, p<0.01).

When considering the population of neurons (Fig. 3A) across the entire sequence, the median spike count for standard tones increased from 0.26 (IQR: 0.84) to 2.54 (IQR: 5.91) spikes per stimulus (976%), while for deviants it increased from 2.00 (IQR: 2.44) to 7.86 (IQR: 12.32) spikes per stimulus (393%) when applying gabazine. The large relative change of the spike count for standards may be due to a floor effect, since the response before application of gabazine was very low, in some cases near zero.

The changes in spike count during the application of gabazine were significant across the population (Friedman test, p<0.01) for both standard and deviant stimuli. Following recovery, spike counts returned to levels that did not differ significantly from control values (Fig. 3B). However, the fact that during the application of gabazine the spike counts evoked by deviant stimuli remained significantly larger (Friedman test, p<0.01) than those evoked by standard stimuli (Fig. 3B) indicates that GABA_A_-mediated inhibition is not the only factor that reduces the discharge evoked by standard, high-probability stimuli.

**Figure 4:**
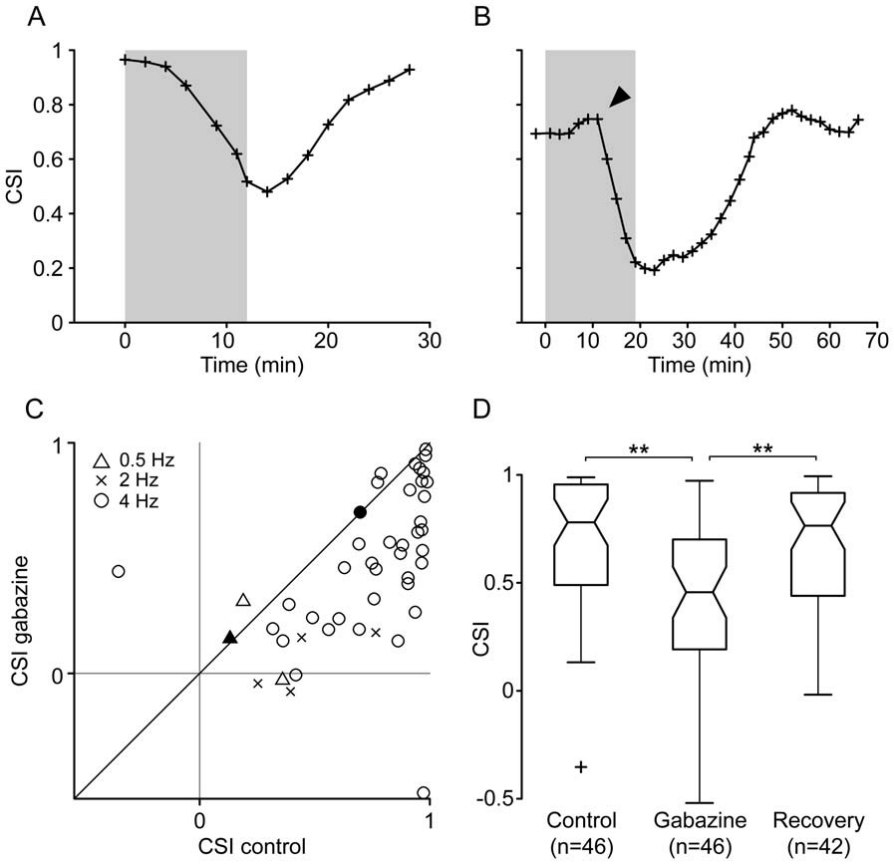
Effect of gabazine on the SSA index. (A, B) Examples of the evolution of CSI during the experiment. The shaded background indicates the application of gabazine, which starts at T = 0. In this and similar figures, the symbols indicate the time at the end of a testing sequence, so all times at or before 0 represent recordings completed before the start of the injection. The neuron shown in (4A) is the same one shown in Fig. 2A–C. The arrowhead in (B) indicates a transient increase in CSI observed in some neurons, probably due to the different time courses with which gabazine affected responses to standards and deviants. (C) In the population of neurons, only 4 showed an increased CSI during the application of gabazine (symbols on the left of the diagonal). The different symbols indicate the repetition rate at which stimuli were presented. The open symbols indicate cases in which there was a significant effect of gabazine (Bootstrapping, 95% c.i.), while the filled symbols indicate cases in which there was no significant effect. (D) Box plot of the CSI values for the population of neurons before, during and after the application of gabazine. The asterisks indicate significant differences (Friedman test, p<0.01).

As illustrated by the two examples shown in Figure 4A,B, CSI decreased significantly for most neurons during the application of gabazine (83%, 38/46; Bootstrapping, 95% c.i.) and gradually returned to control levels after iontophoresis was stopped. However, 4 neurons (9%) showed a significant increase in CSI, and 2 neurons showed no significant change (Fig. 4C). Some neurons (26%; 12/46) showed a small transient increase in CSI at the beginning of gabazine application (Fig. 4B arrowhead). This may have been due to a faster rise in response magnitude for deviant stimuli, as shown in Figure 2E. Across the population, the median CSI decreased significantly (Friedman test, p<0.01) from 0.781 (IQR: 0.45) to 0.457 (IQR: 0.50) during the application of gabazine, and recovered to 0.765 (IQR: 0.47) afterwards (Fig. 4D).

**Figure 5:**
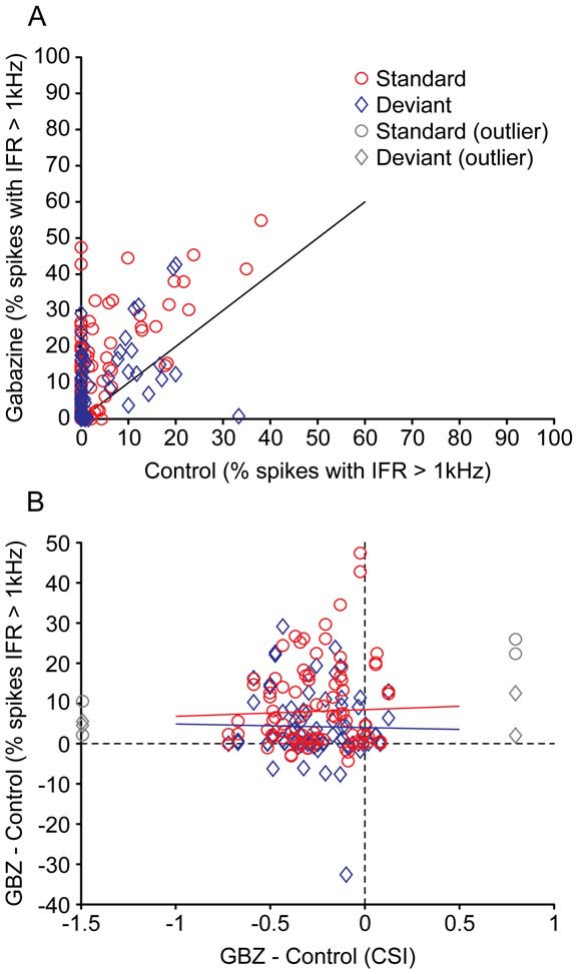
Effect of gabazine on firing rate saturation. (A) Percentage of spikes with an instantaneous firing rate (IFR) faster than 1 kHz. Each data point represents one stimulus (*f_1_* or *f_2_*) at one probability (standard or deviant), for each recorded unit (i.e. each unit produced 4 data points). Blue diamonds indicate standard stimuli; red circles indicate deviant stimuli. (B) The increment in the percentage of IFR faster than 1 kHz during application of gabazine is not correlated with the increment in CSI. As in (A), each data point represents one stimulus at one probability, for each recorded unit. The CSI values are those calculated for the corresponding unit. The lines are the linear regression for the standard (blue) and deviant (red) stimuli. The data points that deviated more than 3 standard deviations from the mean increment in CSI were considered outliers (grey) and were not included in the fitting.

It could be argued that the response to the deviant may have reached a saturating firing rate during the application of gabazine, and that this effect was responsible for the change in CSI. To test whether the increase in firing rate during gabazine application could have caused saturation, we calculated the percentage of spikes that could have fallen within the refractory period of the preceding spike, using as threshold an instantaneous firing rate (IFR) of 1 kHz. Gabazine caused the percentage of IFRs larger than 1 kHz to increase from 2.8% (SD: 6.1) to 7.0% (SD: 9.4) for standard tones, and from 4.0% (SD: 7.7) to 13.2% (SD: 13.8) for deviant tones (Fig. 5A). The potential saturation of firing rate in response to deviant or standard stimuli during gabazine was not correlated with the observed changes in CSI (Fig. 5B), indicating that saturation of the response was not responsible for the observed changes in CSI.

Because GABA is known to play a role in shaping IC neurons' frequency response areas [24], [28], [35], [36] it was necessary to check whether gabazine might have affected the response to one of the test frequencies more than the other. To do this, we calculated the frequency-specific SSA indices (SI(*f_i_*)) for each frequency for each neuron. Analogous to the CSI, the SI(*f_i_*) ranges from −1 to +1, and positive values indicate a larger response when a particular frequency is presented as the deviant. The difference between the SI(*f_i_*) of *f_1_* and *f_2_* was small in the control condition (median: 0.064, IQR: 0.131), but doubled during the application of gabazine (median: 0.123, IQR: 0.187). However, this change was not correlated with the changes in CSI for each neuron (R^2^ = 0.001). This result suggests that gabazine had similar effects on responses to both frequencies, and hence the changes in CSI that we observed were not due to differential effects on neurons' responses to the frequencies tested.

### Effect of gabazine on discharge pattern

**Figure 6:**
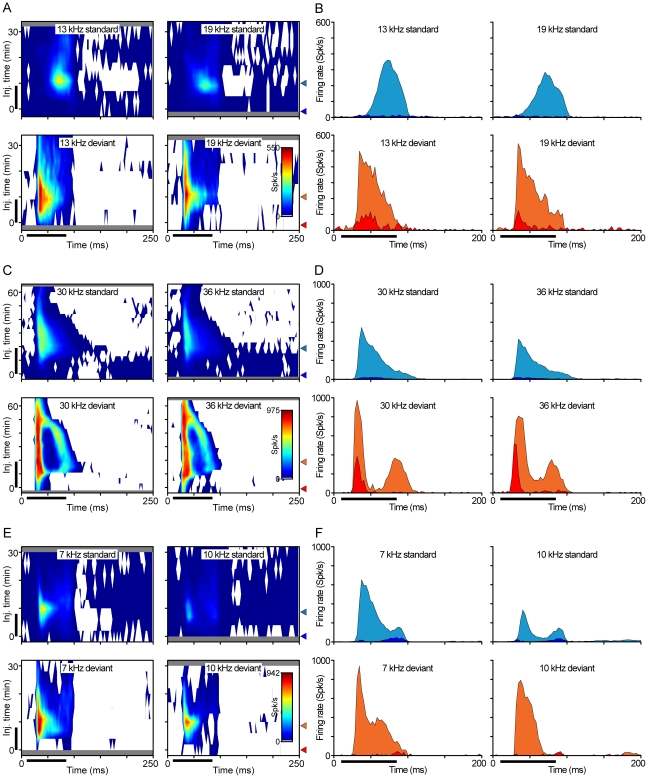
Effect of gabazine on response patterns. (A, C, E) Evolution of the response pattern (abscissa) during the course of the experiment (ordinate), for 3 representative neurons. Each contour plot shows the response of the neuron (color-coded, see scale) for each of the stimuli (columns) and each of the probability conditions (standard, top row; deviant, lower row). The horizontal black bar indicates the duration of the stimulus, while the vertical black bar indicates the application of gabazine. Note that the responses are very similar between frequencies, while they differ depending on the probability of the stimulus. The grey areas in the plots relate to the times when there is no data available for the corresponding frequency and probability, due to the stimulation sequence. (B, D, F) Representative PSTHs (filled areas) for each of the stimuli (columns) and probability conditions (standard, top row; deviant, lower row), before (blue, standard; orange, deviant) and during the application of gabazine (light blue, standard; light red, deviant). The approximate times of these histograms are indicated by triangles on the right of each set of contour plots. Bin size of the PSTHs is 3 ms.

Figure 6 shows examples of changes in discharge pattern during gabazine application. Since we focused on neurons exhibiting high SSA, most of the neurons from which we recorded (85%) were onset responders under control conditions [6], [7]. Gabazine typically caused increased spike counts and lengthened response duration for both standard and deviant sounds. Fig. 6A,C,E shows three examples of how the temporal firing patterns evolved during the application of gabazine for each combination of frequencies and probabilities. In these plots, the abscissa represents the recording time for a single stimulus presentation, where the stimulus duration is indicated by the horizontal black bar below the axis. The ordinate represents the time course of the experiment, where the vertical black bar indicates the duration of the gabazine injection. It is noteworthy that in all cases the changes in firing pattern are very similar for both frequencies, but quite different for the two probability conditions. Fig. 6B,D,F shows representative peristimulus time histograms from the same neurons, comparing their responses before (dark blue, standard; dark red, deviant) and during (light blue, standard; light red, deviant) the application of gabazine, for each combination of frequency and probability. These plots demonstrate that the firing pattern and response latency of these neurons depended mainly on the probability of the stimulus even during gabazine application.

In some neurons (e.g., Fig. 6A,B) the latency of the response to standard tones was much longer than when the same tones were presented as the deviant, and this latency difference was maintained during gabazine application. In 5 neurons gabazine application produced an additional long latency component in the response to deviant stimuli (Fig. 6C,D). This component gradually separated from the original, short latency, onset component. In these neurons, gabazine caused the response to the standard tones to be restored as a sustained response, a pattern very different from that evoked by deviant stimuli. Finally, in a third group of neurons, the differences in firing pattern between deviants and standards were more quantitative than qualitative (Fig. 6E,F), with the discharge patterns evoked by both types of stimuli being similar in time course but of different magnitude. It is noteworthy that in all cases there were large differences in discharge pattern between deviant and standard tones regardless of frequency, indicating that stimulus probability has a larger effect on the patterns of excitation received by these neurons than do physical differences between the stimuli, and that inhibition via GABA_A_ receptors is not responsible for these differences.

### Effect of gabazine on first spike latency

**Figure 7:**
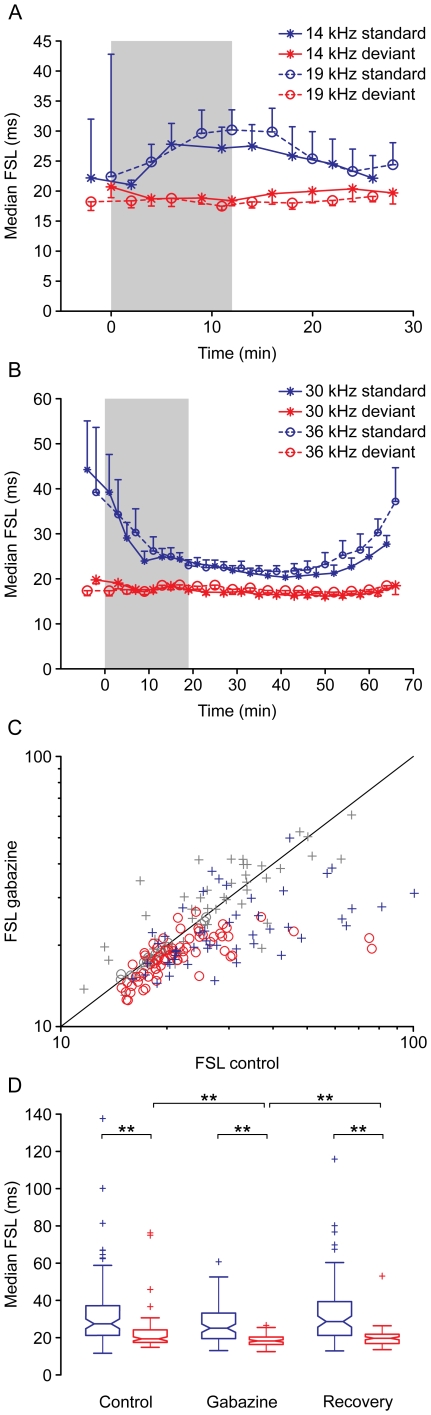
Effect of gabazine on first spike latency. (A, B) Examples of the evolution of FSL during the experiment. While the FSL for deviants tended to decrease by a small but consistent amount during the application of gabazine, the changes of the FSL for standards varied from neuron to neuron, either increasing (A) or decreasing (B). The shaded background indicates the application of gabazine, which starts at T = 0. The error bars represent half the interquartile range. The neuron shown in (7A) is the same one shown in Fig. 2A–C. (C) Median FSL for each stimulus in the control and gabazine conditions. Crosses represent standard stimuli, circles represent deviant stimuli. Responses showing a significant change (Bootstrapping, 95% c.i.) of the FSL are colored in blue (standard) or red (deviant). Changes that are not significant are colored in gray. (D) Box plot showing the median FSL for the population of neurons in the control (Ctrl), gabazine (GBZ) and recovery (Rec) conditions, for deviant (red) and standard (blue) stimuli. The asterisks indicate significant differences (Friedman test, p<0.01).

Gabazine affected the first spike latency (FSL) in response to standard and deviant stimuli in different ways (Fig. 7A,B). Gabazine usually resulted in a small but significant reduction in FSL in response to deviant stimuli (Fig. 7A,B, red lines). However, its effect on responses to standards was more variable. In some neurons, FSL increased (Fig. 7A, blue lines) but in others it decreased (Fig. 7B, blue lines). In both cases the FSL in response to standards before the application of gabazine was highly variable, and this variability was reduced during gabazine application (note the error bars). Across the population, gabazine caused a significant change in FSL for 76% of the deviant stimuli (70/92 frequencies, Fig. 7C, red circles; Bootstrapping, 95% c.i.) but for only 54% of the standard stimuli (50/92 Fig. 7C, blue crosses). In the majority of cases, the change was a decrement of the FSL (41/50 cases for standards, 61/70 cases for deviants). For the population of neurons, in the control condition, the average FSL in response to deviant stimuli was 8.09 ms shorter than the response to standard stimuli (median: 27.43 ms, IQR: 15.90, for standard and 19.34 ms, IQR: 6.71, for deviant stimuli). Application of gabazine (Fig. 7D) caused the average FSL to shorten significantly for deviant tones (median: 18.24 ms, IQR: 3.97, p<0.01), but the change in average FSL for standards was not significant (median: 25.01 ms, IQR: 13.64, p>0.05). The lack of statistical significance may have been due to the larger variability in FSL for standard stimuli both before and during gabazine application (Fig. 7D). The median FSL in response to deviant tones remained significantly shorter than FSL in response to standard tones before, during and after the application of gabazine (Fig. 7D).

### Time course of adaptation

**Figure 8:**
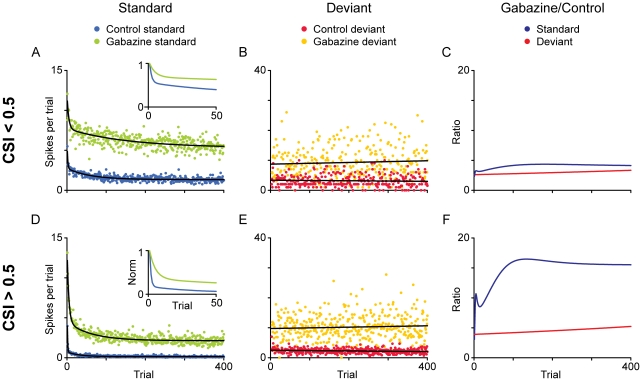
Time course of adaptation. (A–B, D–E) We calculated the average discharge across the population of neurons for each position (trial) in the oddball sequence, separately for standard (A, D) and deviant (B, E) stimuli. We also analyzed separately the minimally adapting neurons (A–C; CSI<0.5 in the control condition) and the highly adapting ones (D–E; CSI>0.5 in the control condition). Then we compared the time course of adaptation in the control condition and during application of gabazine. The data for standard stimuli were fitted by a double exponential function (black lines), and the data for deviant stimuli by a linear function. The insets show a magnified view of the normalized functions during the first 50 trials. Note the different ordinate scales for standard and deviant. (C, F) Ratio of gabazine/control for each type of stimulus during gabazine application.

We analyzed the temporal dynamics of adaptation to the standard and deviant tones across the population during the oddball sequence (Fig. 8). For the standard tones, the time course of adaptation in each condition was fitted by a double exponential function. This function contains both a rapid and a slow decay component, after which the response reaches a steady-state.

The double exponential function closely fit the responses to standard tones (r^2^ = 0.52–0.91) and allowed a detailed analysis of the dynamics of adaptation. In order to avoid possible cancellation of the responses of adapting and non-adapting neurons due to averaging, we analyzed separately the time course for neurons that were highly adapting (CSI>0.5 in the control condition; Fig. 8D) or less adapting (CSI<0.5 in the control condition; Fig. 8A). In the control condition (Fig. 8A,D, blue dots), the rapid component was faster for the highly adapting neurons (τ_r_ = 0.9 trials; 1 trial = 250 ms) than for the less adapting neurons (τ_r_ = 1.5 trials). The application of gabazine (Fig. 8A,D, green dots) made this component slower and virtually eliminated the difference (τ_r_∼4.2 trials for both groups of neurons). The magnitude of the rapid component (*A*_r_) was not affected by the application of gabazine, but it was larger for the highly adapting neurons (*A*_r_≈9.5 spikes per trial, inset 7A) than for the less adapting (*A*_r_≈4.3 spikes per trial, inset 7D). The slow decay component in the control condition was also faster for the highly adapting neurons (τ_s_ = 31.5 trials) than for the less adapting (τ_s_ = 58.8 trials), and in both cases it became notably slower during the application of gabazine (τ_s_ = 61.8 and 153.7 trials, respectively). The magnitude of this component (*A*_s_) in the control condition was smaller for highly adapting neurons (*A*_s_ = 0.6 spikes per trial) than for less adapting neurons (*A*_s_ = 1.4 spikes per trial), because the response of the former was already very low; the application of gabazine equalized this magnitude (*A*_s_ = 2.4 spikes per trial for both highly adapting and less adapting neurons).

The magnitude of the steady-state component (*A*_ss_) indicates that the response was considerably reduced by GABA in the control condition (*A*_ss_ = 0.14 vs. 1.3 spikes per trial, for highly vs. less adapting) as compared to the gabazine condition (*A*_ss_ = 2.1 vs. 5.3 spikes per trial, for highly adapting vs. less adapting), due to the release from inhibition. The magnitude of the steady state was larger for the less adapting neurons than for highly adapting ones, both before and during the application of gabazine.

Adaptation to the deviant stimulus was very low under control conditions, and the population of cells maintained their response to this stimulus through the trials in each condition and during gabazine application. A slight adaptation to the deviant was observed only for the highly adapting units, in the control condition (Fig. 8E, red). Nevertheless, the time course of the deviant stimuli fit poorly to the double exponential function. A linear function gave a more appropriate fit (Fig. 8B,E).

To analyze how each time course was affected by gabazine, we plotted the relative change (gabazine/control) of each function (Fig. 8C,F). The effect of gabazine was roughly linear for the extent of the sequence in the case of the less adapting neurons, both for standard and deviant stimuli. In contrast, for the highly adapting neurons, the effect of gabazine showed a complex shape in which the ratio of gabazine to control increased throughout the first ∼100 trials, after which it became linear. In this part of the function, corresponding to the steady-state, gabazine increased the response about 15 times for highly adapting neurons, while for the less adapting it was only increased about 5 times.

Since gabazine differentially affected the components of the adaptation function, we also calculated the SI(*f_i_*) separately for the beginning of the sequence (first 20 trials) and the end of the sequence (last 100 trials), to analyze the contribution of each portion to the adaptation of the neuron. In the case of the highly adapting neurons, the median SI(*f_i_*) at the beginning of the sequence (0.63) was smaller than at the end of the sequence (0.97). Both values of SI(*f_i_*) dropped by a similar amount (0.28 and 0.23, respectively) during the application of gabazine. The situation for the less adapting neurons was very similar, but all values were smaller, both at the beginning (0.23) and the end (0.43), as well as the amount by which values dropped during the drug condition (0.16 and 0.15, respectively).

## Discussion

This study demonstrates that synaptic inhibition via GABA_A_ receptors modulates the strength of SSA in the IC. We showed that blocking GABA_A_ differentially affected the responses to standard and deviant stimuli presented in an oddball paradigm, reducing the amount of SSA, and partially restoring responses to standard stimuli in neurons with high levels of adaptation. Analysis of the temporal dynamics of SSA showed that the response to standard tones adapted much more slowly during gabazine application than under control conditions, especially during the first repetitions. As would be expected, deviant tones remained virtually non-adapted under all conditions. The present results demonstrate that GABA controls the level of SSA in the IC and contributes primarily to the fast component of adaptation to a high-probability stimulus.

**Figure 9:**
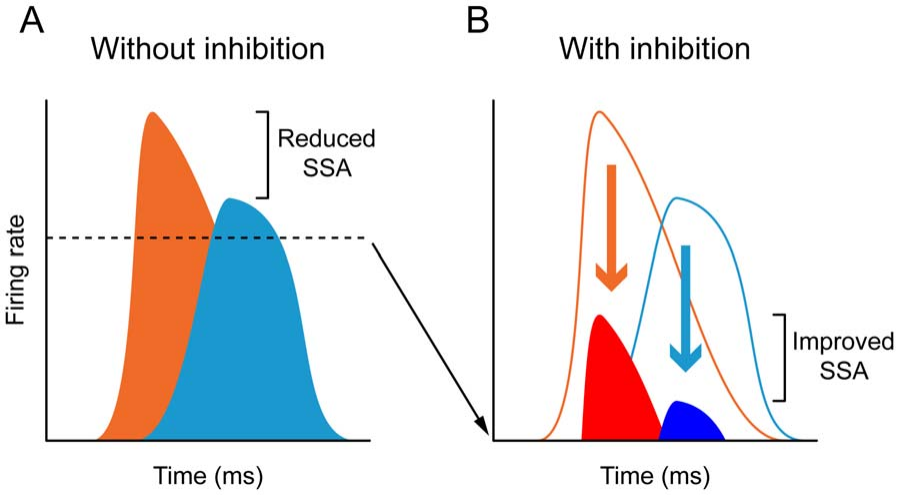
Model of SSA modulation by inhibition. (A) In the absence of inhibition, neurons respond to deviants (orange) and standards (light blue) with high firing rates, and thus the deviant to standard ratio is small. (B) Inhibition reduces the responses to both deviants (red) and standards (dark blue) increasing the deviant to standard ratio and thus enhancing SSA.

Blocking GABA_A_ receptors dramatically increased neurons' firing rate under all conditions and profoundly altered their temporal response patterns. This is in agreement with previous studies that reported a strong influence of GABA on responses of IC neurons [24], [27], [37], [38]. These results suggest that in these cells GABA acts as a gain control system, maintaining the responses within a range such as to maximize the deviant to standard ratio. This is accomplished by rendering excitation due to the standard stimulus subthreshold or near threshold, thus sharpening the contrast between standard and deviant stimuli (Fig. 9), as in the “iceberg effect” [39], [40]. A similar role for GABA_A_-mediated inhibition has been described in this and other systems [25], [41].

We also found that gabazine consistently shortened the FSL in response to deviant tones, but produced more erratic changes in response to standard stimuli. It is possible that very low spike counts in response to standard stimuli under control conditions, with most responses occurring in the first few trials, could have made it difficult to accurately estimate latency in response to the standard, as suggested by the larger dispersion of data before application of gabazine shown in Figure 7A,B. Some previous studies have reported changes in FSL when blocking GABAergic inhibition [42], but others have not found such changes [27], [29], [43]. Methodological differences make it difficult to draw comparisons among those studies or between those studies and ours.

### Technical considerations and limitations

A few points should be addressed before we discuss our results in a functional context. It should be noted that our results are not representative of the whole population of neurons throughout the entire IC, since we deliberately selected those neurons that showed strong adaptation. These strongly-adapting neurons are mainly located in the rostral, dorsal and lateral cortical areas of the IC (RCIC, DCIC and LCIC, respectively), and their physiological characteristics differ in several respects from neurons in the central nucleus (CNIC), which is the largest and most thoroughly studied part of the IC. The neurons in the cortical regions of the IC typically have longer latencies than those in the CNIC, and their response areas tend to be broader, often forming complex mosaics, unlike the V-shaped response areas commonly found in the CNIC [27], [28], [44].

Although the in vivo microiontophoresis technique is a powerful method that allows one to manipulate the neuronal responses at the synaptic level, it has its limitations. We know that the delivery of the drug is close to the recording site, but due to the architecture of the electrodes [27], [28], it is difficult to estimate how widely the drugs spread. Previous studies have shown that iontophoretically delivered drugs can diffuse up to 600 µm [45]. In the rat IC, this range would cover most of the extent of the dendritic arbors of the neurons at the recording site [46]–[48], which can extend up to ∼700 µm in the rostral cortex and up to ∼800 µm in the dorsal cortex [48]. However, we cannot exclude the possibility that the most distant parts of the dendrites were not affected, especially since the tip of the pipette was likely close to the neuronal soma. In fact, a possible explanation for the delayed effect on standard stimuli that we show in Figure 2E is that the GABA_A_ receptors activated by standard stimuli were located farther away from the electrode tip than those activated by deviants, and thus the drug would take more time to reach them. Nevertheless, anatomical evidence would be required to confirm or dismiss this possibility.

It could be argued that the decrement observed in the CSI values during the application of gabazine might have been influenced by the high firing rates in response to the deviant stimuli, leading to a saturation of the response due to depolarization block [29]. However, we showed that the changes in CSI were not correlated with high IFR, which could have led to saturation of the neurons. Therefore, it is safe to conclude that the observed changes in the CSI are genuine and not artifacts of saturated response rates.

The complexity of inhibitory inputs to the IC does not allow us to draw conclusions about the origin of the GABAergic projections affected during the experiments. GABAergic inhibitory projections to the rat IC originate extrinsically from several sources, including bilateral projections from the dorsal nucleus of the lateral lemniscus and ipsilateral projections from the ventral nucleus of the lateral lemniscus [49], [50]. The IC also is an important source of intrinsic and commissural GABAergic inhibition [51], [52].

### Comparison with other studies

In agreement with previous studies, we found that most highly adapting neurons were onset responders [6], [7]. This is related to the fact that onset-responding units are common in the areas where these units are located (RCIC, DCIC and LCIC) [27], [53]. It is also notable that the onset pattern was changed by gabazine. Previously, LeBeau et al. [27] found that the response pattern of onset neurons changed during application of bicuculline, and that this class of neurons was common in the DCIC of the guinea pig. These characteristics are shared by our sample of neurons.

In this study we tested highly adapting neurons, in conditions that are known to elicit high levels of SSA in the IC (i.e. Δ*f* = 0.25–0.37, repetition frequency of 4 Hz, 10% of deviants) and thus the range of CSI values in our sample is quite high (median: 0.78, IQR: 0.45). When testing under similar conditions, some previous studies have reported similar values in the IC. Zhao et al. [20] report a mean CSI ∼0.6 for Δ*f* = 0.37 and 8 stimuli per second, while Malmierca et al. [6] report a mean CSI ∼0.5 for Δ*f* = 0.37 and 4 stimuli per second. Most probably, because of our intentional sampling bias, the neurons in our sample represent the best performers, which could explain the differences between this and more general studies.

### Mechanisms of SSA

A key finding is that SSA was only partially abolished in our experiments. Since our goal was to study the effects of GABA mediated by GABA_A_ receptors, it is apparent that other factors must be involved in the generation of SSA at the level of the IC. These might include GABA_B_- and/or glycine-mediated inhibition, glutamatergic excitation, and neuromodulatory influences. Previous studies have demonstrated that baclofen (a GABA_B_ agonist) produces a significant reduction in the firing rate of IC neurons [27], [54] and that response habituation in the superior colliculus is reduced by blocking GABA_B_ receptors [55]. Response habituation in the superior colliculus can be also modulated by local iontophoretic ejection of either AP5 or CNQX, which block glutamate-mediated activity [56]. Yet another possibility is that SSA in the IC is further modulated by corticofugal projections. These possibilities are not mutually exclusive and, although some of these studies are related to systems other than audition, they clearly demonstrate that adaptation is a complex phenomenon that may result from a combination of multiple factors. Future studies are needed to unveil the role each of them plays on the generation of SSA, in the IC and elsewhere.

It is likely that the mechanisms underlying SSA act at the sites of synaptic input on the IC neuron, and might include synaptic depression and/or facilitation [57]–[59] or inhibition [60] as well as stimulus-specific changes in inhibition [60], [61]. The very different response patterns to deviants and standards observed during blockade of GABA_A_ receptors (Fig. 6) may be an indication that the neurons integrate a different set of excitatory information during each stimulus probability. The longer latency and lower firing rate in response to standard stimuli can be generated if a) the physical stimuli are different enough to travel through at least partially segregated pathways and b) a subset of the excitatory inputs reduce their transmission due to the repetitions of the standard, while the rest of the inputs, not activated by the standard, still respond to the deviant. This mechanism requires confirmation, and similar results can be achieved by inhibitory inputs.

A recent study [62] demonstrated an analog of SSA in cultured networks of cortical neurons. They used an analog of the oddball design, and found a depression in the responses to the standard and an increased response to the deviant. Furthermore, this selective enhancement of responses was abolished by blocking GABAergic inhibitory transmission using bicuculline. We did not see any enhancement of the response to the deviant stimuli in the control condition, which in Eytan et al. [62] was explained by an adaptation of the inhibition, due to the activation of the inhibitory circuits by both standard and deviant stimuli. However, it is possible that different mechanisms operate in the cortex than in the IC.

A recent modeling study has shown that SSA could be explained by excitation followed by a long-lasting wave of inhibition [63]. Wehr and Zador [59], [61] studied the role of GABAergic inhibition in forward masking in the auditory cortex, and found that the inhibitory conductances typically lasted 50–100 ms, so they concluded that, at longer interstimulus intervals, synaptic depression may have a larger influence. The influence of GABAergic inhibition beyond that time frame was limited, but still had some effect on the response to subsequent tones played up to 500 ms afterwards. In the IC, inhibitory conductances are as long-lasting as those in the cortex [33] and SSA can occur at interstimulus intervals up to 2 s (MGB: [5], IC: [6], [20]). This is in agreement with our results and our observation that gabazine produced a larger effect on the fast time constant of adaptation to the standard sound (Fig. 8). Furthermore, a recent study based on a neurocomputational model shows that a combination of adaptation and inhibition (and even Hebbian learning, in the case of familiar stimuli) can result in change detection [64]. A potential scenario to create SSA would be through multiple frequency inputs in which frequently-activated synapses undergo adaptation, possibly through forward masking by presynaptic GABAergic input and rarely-activated ones do not. The possibilities discussed above are obviously not mutually exclusive, and could operate in an interactive fashion.

## Methods

### Surgical procedures

The experiments were performed on 19 adult rats with body weights between 115 and 310 g. All experiments were performed at the University of Salamanca with the approval of, and using methods conforming to the standards of, the University of Salamanca Animal Care Committee.

Surgical anesthesia was induced and maintained with urethane (1.5 g/kg, i.p.), with supplementary doses (0.5 g/kg, i.p.) given as needed. Urethane was chosen as an anesthetic because its effects on multiple aspects of neural activity including inhibition and spontaneous firing are known to be less than those of barbiturates and other anesthetic drugs [65]. The animals were connected to a ventilator (SAR-830/P) and the expired CO_2_ was monitored using a capnograph (Capstar-100). The trachea was cannulated, and atropine sulfate (0.05 mg/kg, s.c.) was administered to reduce bronchial secretions. Body temperature was maintained at 38±1°C. Details of surgical preparation were as described previously [7], [44], [66]–[70]. The animal was placed in a stereotaxic frame in which the ear bars were replaced by hollow specula that accommodated a sound delivery system. A craniotomy was performed to expose the cerebral cortex and the cerebellum overlying the IC.

### Electrophysiological recording and pharmacology

A tungsten electrode (1–2 MΩ) [71] was lowered through the cortex using a Burleigh microdrive, and used to record extracellular single unit responses in the IC. Action potentials were amplified (×10,000), band-pass filtered (Butterworth filter; 0.3–8 kHz) and discriminated using TDT System 2 hardware (Tucker-Davis Technologies). Single units were identified by the constant shape and magnitude of the spikes, which was monitored throughout the experiment. The threshold for spike discrimination was adjusted individually for each unit. To avoid recording multiunit activity we tried to keep a SNR>5. In some complicated cases we programmed the spike discriminator (TDT SD1) to detect additional spike characteristics such as peaks and valleys. Figure 1 shows a typical example of spike waveforms before, during, and after gabazine application.

Spike times were logged on a computer, where stimulus generation and online data visualization were controlled with custom software [72]. The recording electrode was attached to a multibarrel glass pipette, so that the tip of the recording electrode protruded 10–20 µm. The glass pipette consisted of five barrels in H-configuration, with the tip broken to a diameter of 10–25 µm). One of the barrels was filled with saline for current compensation (1 M NaCl), while the others were filled with 20 mM gabazine (SR-95531) that was prepared in distilled water and the pH was adjusted to 4.0. Gabazine is a selective antagonist of GABA_A_ receptors with reduced side effects, in contrast with bicuculline, the other typical antagonist of GABA_A_ receptors, which apart from being less selective has unwanted effects on calcium-dependent potassium channels [73]. The drug was retained by applying a 15 nA current, and was ejected when required, typically using 40–50 nA currents (Neurophore BH-2 System, Harvard Apparatus). In the data presented here, current alone at levels comparable to drug injection currents had no effect on neural activity, thereby ruling out current artifact as a contaminating variable. To test for the saturation of neuronal responses due to action of gabazine [74], we recorded from 10 neurons using several different ejection currents (ranging from 30 nA up to 100 nA). In 9 of these neurons, larger currents always produced increased firing rates. In one neuron all of the currents tested (50–60 nA) reduced the response of the neuron. The resistance of each drug barrel was checked before entering the IC and also at the end of the experiment, to make sure that no barrels were blocked. The ejection was continued until a clear increment of the response was observed. On average, the duration of the drug ejection was 18 min, but it ranged from 7 to 42 minutes. Complete recovery (which in some cases took up to 57 min) was observed in 28 neurons (61%). The rest of the neurons could not be held for enough time to record a complete recovery, but in most cases a partial recovery or a trend back toward control values was observed.

### Acoustic delivery and stimulus presentation paradigms

Stimuli were delivered through a sealed acoustic system [75], [76] using two electrostatic loudspeakers (model EC1, Tucker-Davis Technologies). The output of the system at each ear was calibrated *in situ* using a ¼ inch condenser microphone (model 4136, Brüel & Kjær) and a DI-2200 spectrum analyzer (Diagnostic Instruments). The maximum output of the system was flat from 0.5 to 4 kHz (∼110±7 dB SPL), from 4.5 to 14.5 kHz (∼90±6 dB SPL) and from 15.5 to 40 kHz (±95±7 dB SPL), presenting a notch at 15 kHz. The highest frequency produced by this system was limited to 40 kHz. The second and third harmonic components in the signal were >40 dB below the level of the fundamental at the highest output level [6], [69]. The stimuli were presented contralaterally to the recording side. Search stimuli included white noise and pure tones whose frequency was changed manually to reduce the adaptation of the neurons so that we would not miss their responses. As we were interested in the properties of those neurons that experience SSA, we did not record from those neurons that did not adapt to repetitive stimuli.

The experimental stimuli were pure tones in the range 0.5–40 kHz, with a duration of 75 ms, including 5 ms rise/fall ramps. At the beginning of the experiment, the frequency response area (FRA) of the cell, i.e. the combination of frequencies and intensities capable of evoking a response, was obtained automatically using a randomized paradigm that presented tones between 0.5 and 40 kHz in 25 logarithmic steps, with intensities spaced by 10 dB steps, at a rate of 4/s. This allowed us to choose pairs of frequencies (*f_1_* and *f_2_*) that elicited a similar firing rate at the same level (10–40 dB above threshold), which were then presented in an oddball paradigm [6], [8], [77], [78]. The separation of the frequencies was typically 0.36–0.53 octaves, which corresponds to Δ*f* = 0.25–0.37 [8], [78]. We presented a train of 400 stimuli containing both frequencies in a probabilistic manner. One frequency (*f_1_*) was presented as the standard (i.e., high probability within the sequence, 90%); interspersed randomly among the standards were the deviant stimuli (i.e., low probability, 10%) at the second frequency (*f_2_*). The repetition rate of the stimuli was typically 4/s, the most effective in eliciting SSA in the IC [6], but a subset of neurons were tested at lower repetition rates, up to 0.5/s. After obtaining one data set, the relative probabilities of the two stimuli were reversed, with *f_2_* as the standard and *f_1_* as the deviant. The responses to the standard stimulus and deviant stimulus were normalized to account for the different number of presentations in each condition, because of the different probabilities. The original and the reverse sequence were alternated during the whole course of the experiment during which time we obtained the responses of the cell under control conditions, at different times during drug application and during recovery.

### Data analysis and statistics

The spike times evoked by the different stimuli were stored and used to calculate neurometrics such as the response magnitude (spikes per trial), first spike latency (FSL, relative to the onset of the stimulus), instantaneous firing rate (IFR) or SSA indices (see below). For all these calculations, a time window was applied to each unit that included the whole evoked response, avoiding the spontaneous activity when present. Data representative of the control condition were taken before the beginning of the drug application. Data representative of the gabazine condition were taken from the time point with the largest departure from the control values, during the application of drug or afterwards. Data representative of the recovery were obtained whenever we were able to hold the neuron for enough time and the responses returned to values close to the control. When those conditions were met, we used the last recorded data in the experiment as representative of recovery. These representative values were used to test the significance of the changes produced by gabazine on each neuron, using the bootstrapping method with a 95% confidence interval.

To analyze the effect of gabazine in the population of neurons recorded, we calculated the distributions of the mean response magnitude (in spikes per trial), CSI and median FSL, using values representative of the control, effect and recovery conditions, and plotted them as boxplots (Fig. 3B, 4C, 7C). We then compared the groups, looking for statistical differences using the Friedman test, adjusted for multiple comparisons by the Bonferroni method.

The instantaneous firing rates (IFR, Fig. 5) were computed as the inverse of the individual interspike intervals, in seconds.

To quantify the amount of SSA that occurred, we calculated two different forms of the SSA index using the method described by Ulanovsky et al. [8], [78]. One was the frequency-specific index SI(*f_i_*), where i = 1 or 2, defined for each frequency *f_i_* as SI(*f_i_*) = [d(*f_i_*)−s(*f_i_*)]/[d(*f_i_*)+s(*f_i_*)], where d(*f_i_*) and s(*f_i_*) are responses (as normalized spike counts) to frequency *f_i_* when it was deviant or standard, respectively. The other SSA index was the common SSA index (CSI) defined as CSI = [d(*f_1_*)+d(*f_2_*)−s(*f_1_*)−s(*f_2_*)]/[d(*f_1_*)+d(*f_2_*)+s(*f_1_*)+s(*f_2_*)], where d(*f_i_*) and s(*f_i_*) are responses to each frequency *f_1_* or *f_2_* when they were the deviant or standard stimuli, respectively. These indices reflect the extent to which the response of a cell to the standard sound was suppressed and/or the response to the deviant was enhanced. The possible range of values for both indices is from −1 to +1, being positive if the response to the deviant stimulus was greater, and negative if the response to the standard stimulus was greater. The median CSI in control condition was 0.78 (IQR = 0.45), because recordings were mostly from the cortical regions of the IC, where neurons show the highest degree of SSA [6], [7].

The contour plots in Figure 6A,C,E represent the variation of the peristimulus time histogram (PSTH) during the course of the experiment. We obtained the PSTH (3 ms bin size) for each frequency and probability value, at different times before, during and after the application of gabazine. Then we used the *contourf* function in MATLAB to plot the time relative to the stimulus onset on the abscissa, the time relative to the injection start on the ordinate and the spike counts in each bin as a color code.

To characterize the time course of SSA in the oddball design, the responses to the *k* standard trials and the (400−*k*) deviant trials were combined by their order of presentation in the sequence, averaged across all neurons, and then plotted at their original 400-trial-long time scale (see Fig. 8). For the standard trial we performed a nonlinear least-square fit to this population mean curve to find the best-fitting double exponential function as follows: *f(t)* = *A*_ss_+*A*_r_ · *e* ^−*t*/τ(r)^+*A*_s_ · *e* ^−*t*/τ(s)^, where *A*_ss_, *A*_r_ and *A*_s_ are the magnitudes of the steady state, and the rapid and slow components, respectively, and τ_r_ and τ_s_ are the time constants of the rapid and slow components. The deviant trials were fitted to a linear function *f(t)* = *a*+*bt*.

### Histological verification of recording sites

Neuron location in the IC was based on stereotaxic coordinates, physiological criteria of tonotopic organization, response properties, and histological verification using electrolytic lesions (5–10 µA for 5–10 s) to mark recording sites [7], [44], [46], [66], [69], [70], [79]. At the end of each experiment, the animal was given a lethal dose of sodium pentobarbital and perfused transcardially under deep surgical anesthesia with PBS (0.5% NaNO_3_ in PBS) followed by fixative (a mixture of 1% paraformaldehyde and 1% glutaraldehyde in rat Ringer's solution). After fixation and dissection, the brain tissue was cryoprotected in 30% sucrose and sectioned on a freezing microtome in the transverse or sagittal planes into 40- to 50-µm-thick sections. Sections were stained with 0.1% thionin blue to facilitate identification of cytoarchitectural boundaries [46], [47], [80]. All of the neurons were located in the dorsal and lateral cortices of the IC.
