# Correction: GABA_A_-Mediated Inhibition Modulates Stimulus-Specific Adaptation in the Inferior Colliculus

**DOI:** 10.1371/annotation/883c90e9-2108-449f-a652-2cbf25ba6456

**Published:** 2012-08-15

**Authors:** David Pérez-González, Olga Hernández, Ellen Covey, Manuel S. Malmierca

There was an error in the Funding statement. The correct Funding is:

Financial support was provided by the Spanish MEC (BFU2009-07286), Spanish MICINN (EUI2009-04083) in the frame of the ERA-NET NEURON, and JCYL-UE (GR221) to Dr. Malmierca and the NSF (IOS-0719295) to Dr. Covey. The funders had no role in study design, data collection and analysis, decision to publish, or preparation of the manuscript.

